# The gastric double-stripe sign of chronic mesenteric ischemia: A case report

**DOI:** 10.1097/MD.0000000000032842

**Published:** 2023-02-03

**Authors:** Hiroyuki Yamamoto, Toru Hashimoto

**Affiliations:** a Department of Cardiovascular Medicine, Narita-Tomisato Tokushukai Hospital, Chiba, Japan.

**Keywords:** chronic mesenteric ischemia, Doppler ultrasound, esophagogastroduodenoscopy, gastric double-stripe sign, refractory gastric ulcer

## Abstract

Atherosclerotic chronic mesenteric ischemia (CMI) is caused by stenosis or occlusion of mesenteric arteries and can progress to potentially life-threatening acute mesenteric ischemia if left untreated. However, correct diagnosis and timely treatment remain a challenge due to a lack of awareness, variable symptoms, and inconclusive diagnostic testing.

**Patient concerns:**

An 82-year-old man with multiple cardiovascular risk factors, including chronic kidney disease, presented with dyspnea, anorexia, and oliguria. Laboratory results revealed severe renal dysfunction (raised serum blood urea nitrogen of 83.8 mg/dL, serum creatinine levels of 8.20 mg/dL, and decreased estimated glomerular filtration rate of 5.5 mL/min/1.73 m^2^), hypoalbuminemia, and moderate anemia. A provisional diagnosis of acute exacerbation of chronic kidney disease was made and the patient required tentative intermittent hemodialysis, received blood transfusions, and was eventually placed on maintenance hemodialysis. However, the patient’s symptoms did not improve. Esophagogastroduodenoscopy (EGD) revealed longitudinal gastric ulcers on the anterior and posterior walls of the stomach, which were named “a gastric double-stripe sign” because the lesions corresponded to the watershed areas of the stomach. No *Helicobacter pylori* infection or malignancy was identified, and increasing the dose of lansoprazole had no beneficial effects. Doppler ultrasound revealed high peak systolic velocity (270 cm/s) of the celiac artery (CA), suggesting CA stenosis, which was confirmed by magnetic resonance angiography.

**Diagnosis:**

Final diagnosis of CMI was made based on patient’s symptoms, EGD findings, Doppler ultrasound, and magnetic resonance angiography.

**Interventions:**

Endovascular revascularization for CA stenosis was performed.

**Outcomes:**

The patient obtained symptomatic relief concomitant with the resolution of the gastric ulcers. The post-procedural course of the patient was uneventful and he remained healthy at the 1-year follow-up.

**Lessons:**

This is the first case of CMI with EGD finding of a gastric double-stripe sign specific for gastric ischemia. This case highlights the clinical importance of this endoscopic finding in patients with suspected atherosclerotic CMI.

## 1. Introduction

Chronic mesenteric ischemia (CMI) with atherosclerotic etiology is a serious peripheral vascular disease that results from an inability of the blood supply to meet the metabolic demands of the visceral organs; it can lead to fatal acute mesenteric ischemia if left untreated.^[1]^ Although the incidence of CMI was considered low thus far, a recent study with a prospective database analyzing 385 patients with CMI demonstrated that the mean incidence of atherosclerotic CMI is 7.3 (95% confidence interval: 4.6–11.3) per 100,000 inhabitants, and it further increases with age,^[2]^ suggesting that CMI occurs more often than previously thought. CMI is most frequently diagnosed in women >60 years of age with other coexisting arteriosclerotic diseases.^[3]^ However, due to highly variable symptoms and inconclusive diagnostic testing, an accurate diagnosis is easily missed or delayed, and timely treatment is often missed.

## 2. Case presentation

An 82-year-old man was admitted to our hospital with dyspnea, anorexia, and oliguria. Although the patient complained of postprandial abdominal discomfort, nausea, and anorexia for 2 months, he had a weight gain of 5.0 kg. The cardiovascular risk factors included hypertension, hyperlipidemia, diabetes, and stage 3B chronic kidney disease (CKD). The patient also had a history of stroke and coronary artery bypass graft surgery. The patient had no history of smoking, alcohol consumption, or illicit substance use. He denied taking non-steroidal anti-inflammatory drugs (NSAIDs). Pre-admission medication included carvedilol (1.25 mg twice daily), losartan (25 mg/day), aspirin (100 mg/day), lansoprazole (15 mg/day), rosuvastatin (5 mg/day), linagliptin (5 mg/day), and voglibose (0.3 mg 3 times daily before each meal). On admission, the patient weighed 60 kg. His vital signs were as follows: blood pressure, 110/67 mm Hg; heart rate, 70 beats/min; and oxygen saturation, 92% in ambient air. Physical examination revealed diminished breath sounds from both the lower lungs and marked generalized edema. Chest radiography revealed cardiomegaly with mild pulmonary congestion and bilateral pleural effusions. Echocardiography demonstrated preserved left ventricular systolic function, with an ejection fraction of 54%. Laboratory tests revealed elevated levels of serum blood urea nitrogen (83.8 mg/dL, reference: 8–20 mg/dL), creatinine (8.20 mg/dL, reference: 0.65–1.07 mg/dL), C-reactive protein (0.86 mg/dL, normal <0.3 mg/dL), and an estimated glomerular filtration rate of 5.5 mL/min/1.73 m^2^, whereas serum total protein (6.2 g/dL, reference: 6.6–8.1 g/dL) and albumin (2.3 g/dL, reference: 4.1–5.1 g/dL) levels were decreased. Moderate anemia was observed, with decreased red blood cells (271 × 10^4^/μL, reference: 435–555 × 10^4^/μL), hemoglobin level (7.6 g/dL, reference: 13.7–16.8 g/dL), hematocrit level (23.0%, reference: 40.7–50.1%), and mean corpuscular volume (85.2 fL, reference: 85–99 fL), suggestive of normocytic anemia. Hepatic and thyroid functions were within normal ranges. Two sets of blood cultures and a urine culture remained sterile.

**Figure 1.**
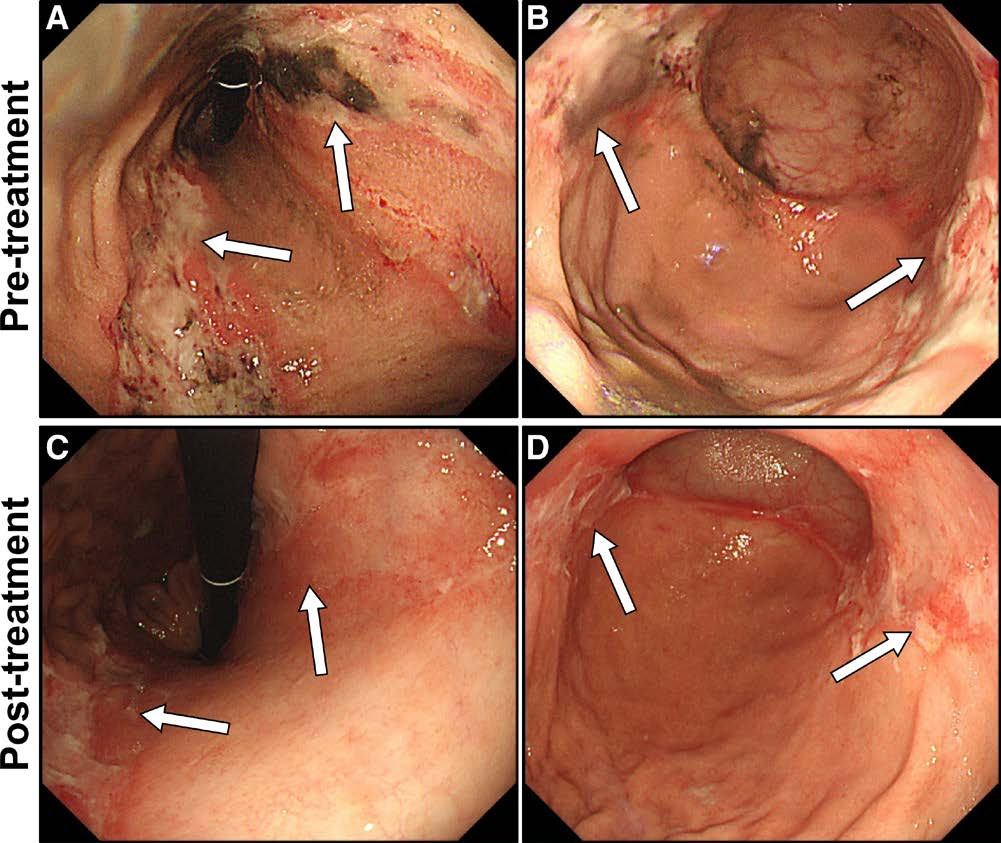
Endoscopic images before and after endovascular treatment. (A, C, gastric angularis view; B, D, distal gastric corpus and antrum view). (A and B) Endoscopic images reveal each linear necrotic ulceration extending longitudinally from the body to the fundus on both the anterior and posterior gastric walls with surrounding hyperemic mucosa (arrows). (C and D) Follow-up endoscopic images 1 month after endovascular treatment reveal significant resolution in the gastric lesions that have been replaced by ulcer scars (arrows).

**Figure 2.**
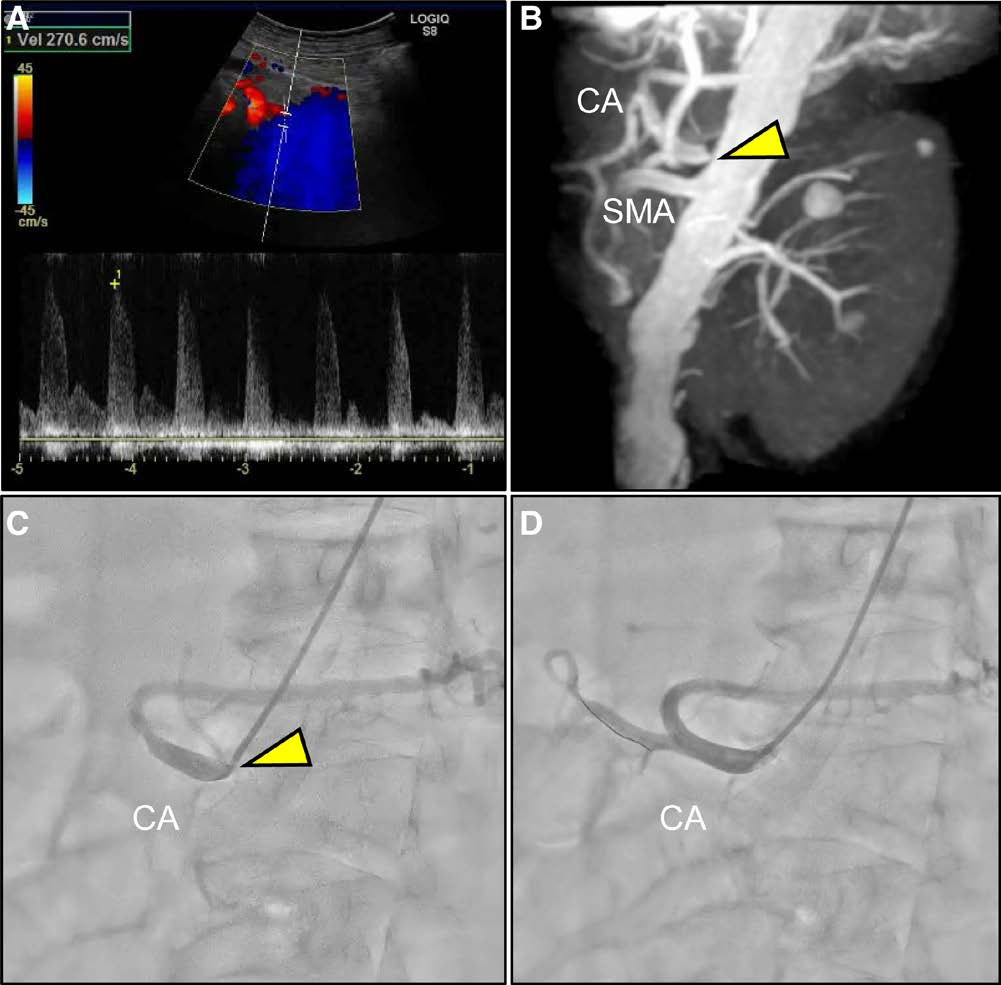
Multimodality imaging of stenosis of the CA. (A) Duplex ultrasonography of the CA reveals peak systolic velocity of 270 cm/s. (B) Noncontrast-enhanced magnetic resonance angiography in sagittal view reveals high-grade stenosis of the celiac trunk origins at the aorta (arrowhead). Selective angiography of the CA in left anterior oblique view (C) before and (D) after endovascular treatment. Note the subtotal stenosis at the origin of the celiac trunk (arrowhead). CA = celiac artery, SMA = superior mesenteric artery.

Based on these findings, a diagnosis of acute exacerbation on CKD was made. Oral losartan was discontinued after admission. The patient required tentative intermittent hemodialysis and received red blood cell transfusions, following which the hemoglobin level increased to 9 g/dL. However, the patient’s renal dysfunction did not improve. He was eventually placed on maintenance hemodialysis. His persistent anorexia, nausea, and abdominal discomfort prompted further evaluation. Abdominal examination revealed mild epigastric tenderness without guarding. Esophagogastroduodenoscopy (EGD) revealed longitudinal gastric ulcers (GUs) along the midline of both the anterior and posterior walls of the stomach, suggestive of a watershed area of the stomach, but no *Helicobacter pylori* bacteria or malignancy was identified (Fig. 1A and B). Lansoprazole dosage was increased (30 mg once daily) but it did not improve his symptoms, and the 1-month follow-up EGD did not show any improvement in the GUs. The endoscopic findings suggestive of vascular etiology prompted us to perform further evaluations. Although the abdominal bruit was unremarkable, a mesenteric Doppler ultrasound (DUS) revealed that the peak systolic velocities of the trunk of the celiac artery (CA) and superior mesenteric artery (SMA) were 270 cm/s and 155 cm/s, respectively (Fig. 2A), strongly suggesting CA stenosis. Abdominal magnetic resonance angiography confirmed severe CA ostial stenosis (Fig. 2B). Subsequently, percutaneous endovascular revascularization was performed using the left transbrachial approach (Fig. 2C and D). After systemic heparinization was achieved, a 6F MPA Vista Brite Tip guiding catheter (Cordis, Hialeah, FL) was advanced into the abdominal aorta over the previously placed guidewire and a 4F JR4.0 catheter to engage at the origin of the CA. The origin of the CA was dilated with a 5 mm angioplasty balloon, and a 6 mm × 14 mm Express Vascular SD stent (Boston Scientific, Marlborough, MA) was deployed. A gradient of 30 mm Hg across the lesion observed preoperatively, resolved postoperatively. The patency of the CA was confirmed with a final angiography. Dual antiplatelet therapy was initiated for 3 months, followed by single antiplatelet therapy.

One month later, a repeat EGD after endovascular revascularization revealed significant improvement in the GUs (Fig. 1C and D). Based on these findings and the patient’s clinical course, a final diagnosis of CMI was made. At the 3-month follow-up after admission, he reported complete resolution of all his symptoms and was transferred to a nursing home for rehabilitation because of advanced disuse atrophy. At the 1-year follow-up after discharge, the patient was asymptomatic, with no recurrence.

## 3. Discussion

Because of multiple arteries supplying the stomach and enrichment of the submucosal vascular plexus, the stomach is considered to be resistant to ischemia. Therefore, chronic gastric ischemia is relatively rare in patients with CMI. Gastric ischemia is mainly attributed to diffused or localized inadequate blood flow to the stomach. Its etiologies include systemic hypoperfusion (shock and sepsis) and splanchnic vessel hypoperfusion (vessel stenosis, disseminated thromboembolism, gastric volvulus, vasculitis, vasoconstriction, and endoscopic therapeutic interventions).^[4]^ Common symptoms are often misdiagnosed as other gastrointestinal etiologies, such as gastric enteritis or peptic ulcers. Herein, we describe a patient with CMI caused by CA stenosis who presented with refractory GUs and was successfully treated with endovascular revascularization. Our case offers 3 important clinical implications.

First, our patient presented with atypical signs of CMI and refractory GUs without weight loss.

The presenting symptoms of CMI vary and include postprandial pain, adapted eating pattern, weight loss, nausea, vomiting, chronic diarrhea, or constipation.^[5]^ In particular, ongoing gastric ischemia results in full-thickness hemorrhagic necrosis, gastrointestinal bleeding, and shock in advanced cases.^[6]^ The classic clinical triad of CMI, including postprandial pain, abdominal bruit, and weight loss, is of limited value for detecting patients with a CMI of 22%.^[7]^ The probability of CMI was 60% in the presence of all 4 symptoms characteristic of CMI (postprandial pain, chronic diarrhea, altered eating patterns, and weight loss), and it was 13% even when all the symptoms were absent.^[5]^ Our patient presented with postprandial abdominal discomfort without weight loss. Given that anorexia caused by CMI presumably triggered acute exacerbation of CKD in our case, subsequent volume overload might have led to weight gain and eventually obscured one of the characteristic clinical features of CMI, namely weight loss. Therefore, this case underscores the clinical importance of considering CMI even in the absence of weight loss.

Second, in our case, CMI manifested as a gastric double-stripe sign on EGD.

EGD is an important screening test in patients with CMI to rule out malignancy and ulcers associated with *H. pylori* infection or NSAIDs. It is believed that there are no specific EGD findings of CMI^[8]^; edema, erythema, atrophy of the gastric mucosa, and gastric/duodenal ulcers unrelated to *H. pylori* and NSAIDs are documented in limited cases with CMI. Furthermore, a prospective study analyzing 56 patients with CMI compared to 26 patients without CMI revealed that histological examination of the biopsy samples yielded no significant value in detecting CMI.^[9]^ This indicates that normal EGD findings do not always exclude CMI. In addition, several large retrospective observational studies have revealed that in EGD, lesions of CMI are often accompanied by multiple or diffuse large ulcers.^[4]^ The most frequently affected areas were the greater curvature of the body (66%) and the fundus along the posterior wall of the stomach (33%),^[6]^ corresponding to anastomosis of the arteries of the stomach between the lesser curvature and greater curvature along the anterior and posterior walls of the stomach. Therefore, when patients with CMI present with GUs, the distribution and morphology of the GUs can presumably be an important clue to suspect CMI. Interestingly, in our case, EGD revealed double longitudinal ulcers extending from the body to the fundus on the anterior and posterior walls of the stomach, strongly suggesting watershed areas of the stomach, all of which resolved following endovascular treatment. Because of its novelty, as per our knowledge, we named these unique double longitudinal ulcers seen on EGD “the gastric double-stripe sign” in analogy to “the colon single-stripe sign” indicating a single linear ulcer running along the longitudinal axis of the colon at the watershed areas of the colon.^[10]^ Further analysis is warranted to validate the gastric double-stripe sign for detecting CMI.

Finally, DUS was also useful for detecting significant stenosis of the CA in our case.

A larger study using DUS demonstrated that peak systolic velocity values can offer high diagnostic accuracy for detecting significant mesenteric artery stenosis of >70% (CA, 80% sensitivity, 90% specificity; SMA, 90% sensitivity, 92% specificity).^[11]^ Although CMI is most frequent in symptomatic patients with atherosclerotic multivessel disease, even single-vessel disease may cause CMI (5%).^[12]^ Interestingly, asymptomatic atherosclerotic mesenteric artery stenoses are often observed in the general population: the prevalence of significant atherosclerotic obstructive visceral artery lesions (CA or SMA) identified by DUS was 18% in asymptomatic elderly patients >65 years, and it increases with age. In particular, 1-vessel lesions are observed more frequently in CA than in SMA (81% vs 19%).^[13]^ Therefore, the presence of single-vessel disease does not necessarily indicate CMI. Although endovascular or open surgical revascularization is widely accepted for symptomatic CMI patients with multivessel disease, the same treatment for those with a single vessel remains to be established because of the presence of rich mesenteric collateral circulation. However, most symptomatic CMI patients with single-vessel disease that were carefully selected by a multidisciplinary team achieved a significant symptomatic relief of 73% after treatment.^[14]^ Similarly, in our case, an accurate diagnosis of CMI was established based on the evidence that the single-vessel disease of CA stenosis was resolved by endovascular mesenteric revascularization with concomitant recovery of the refractory GUs and symptomatic relief. Further prospective studies are needed to determine the advantages and disadvantages of revascularization in symptomatic CMI with single-vessel disease. A large meta-analysis including 100 observational studies reported that an endovascular approach is associated with lower perioperative complication rates, shorter hospital stays, and lower costs compared to those of a surgical approach.^[15]^ Although an endovascular approach has a higher restenosis rate, it has fewer perioperative complications, and is thus comparable to a surgical approach in terms of long-term mortality. Our patient was an elderly, high-risk patient with CA ostial lesions; therefore, we opted for less invasive endovascular treatment. However, highly tortuous aorta or iliac arteries, small-diameter vessels, heavily calcified stenosis, or long-segment occlusion are associated with lower technical success rates, higher procedural complications, and lower patency rates.^[11]^ Surgical treatment is another preferred option for such cases. An individualized treatment approach, based on the patient’s background, is desirable.

In conclusion, we describe a case of a patient with CMI caused by CA stenosis, presenting with refractory GUs that was successfully treated with endovascular revascularization. A gastric double-stripe sign on EGD and DUS aided in the diagnosis of atherosclerotic CMI. Symptomatic CMI may progress to potentially fatal sequelae, such as gastric or bowel necrosis or perforation, without prompt mesenteric artery revascularization. Therefore, clinicians should not underestimate the importance of this disease; they should consider CMI as a cause of unexplained refractory GU in any patient with atherosclerotic burden and perform EGD and mesenteric vascular screening with DUS.

## Author contributions

**Conceptualization:** Hiroyuki Yamamoto.

**Data curation:** Hiroyuki Yamamoto, Toru Hashimoto.

**Investigation:** Hiroyuki Yamamoto.

**Supervision:** Toru Hashimoto.

**Validation:** Toru Hashimoto.

**Writing – original draft:** Hiroyuki Yamamoto.
